# Pressure Evolution of Ultrafast Photocarrier Dynamics and Electron–Phonon Coupling in FeTe_0.5_Se_0.5_

**DOI:** 10.3390/ma15238467

**Published:** 2022-11-28

**Authors:** Muyun Li, Yan Zhou, Kai Zhang, Guangyong Xu, Genda Gu, Fuhai Su, Xiaojia Chen

**Affiliations:** 1 Center for High Pressure Science and Technology Advanced Research, Shanghai 201203, China; 2 Shanghai Insititude of Space Power Source, Shanghai 200245, China; 3 Key Laboratory of Materials Physics, Institute of Solid State Physics, HFIPS, Chinese Academy of Sciences, Hefei 230031, China; 4 NIST Center for Neutron Research, National Institute of Standards and Technology, Gaithersburg, MD 20899, USA; 5 Condensed Matter Physics & Materials Science Division, Brookhaven National Laboratory, Upton, NY 11973, USA; 6 School of Science, Harbin Institute of Technology, Shenzhen 518055, China

**Keywords:** iron chalcogenides, phonons, Raman scattering, femtosecond

## Abstract

Understanding the coupling between electrons and phonons in iron chalcogenides FeTe${}_{x}$Se${}_{1-x}$ has remained a critical but arduous project in recent decades. The direct observation of the electron–phonon coupling effect through electron dynamics and vibrational properties has been lacking. Here, we report the first pressure-dependent ultrafast photocarrier dynamics and Raman scattering studies on an iron chalcogenide FeTe${}_{0.5}$Se${}_{0.5}$ to explore the interaction between electrons and phonons in this unconventional superconductor. The lifetime of the excited electrons evidently decreases as the pressure increases from 0 to 2.2 GPa, and then increases with further compression. The vibrational properties of the $A_{1g}$ phonon mode exhibit similar behavior, with a pronounced frequency reduction appearing at approximately 2.3 GPa. The dual evidence reveals the enhanced electron–phonon coupling strength with pressure in FeTe${}_{0.5}$Se${}_{0.5}$. Our results give an insight into the role of the electron–phonon coupling effect in iron-based superconductors.

## 1. Introduction

The iron chalcogenides (FeTe${}_{x}$Se${}_{1-x}$) have ignited the research field of unconventional superconductors because of many novel properties such as the high-temperature superconductivity [1,2,3], the high optical absorption coefficient for fabricating the photo-sensing device [4,5,6], and the high superheating field for realizing radio-frequency cavities [7]. Although constructed with the typical layered structure like other iron-based [8,9] and cuprate superconductors [10,11], iron chalcogenides possess the simplest crystal structure, making them much easier to produce [1]. The crystallized structure with a high superheating field allows iron chalcogenides to have much longer wavelengths, compared with the glassed chalcogenides [12,13,14,15], to be coated as a multilayer structure for realizing superconducting radio-frequency cavities [7]. Combining superconducting properties with acousto-optical properties in iron chalcogenides holds attractive application prospects. During the last decade, many high-quality iron chalcogenides such as polycrystal [1,2,16,17,18], single-crystal [19,20,21,22], and two-dimensional samples [23,24,25] were successfully synthesized and systematically studied. Regardless of the abundant research, the pairing mechanism in the superconductivity of iron chalcogenides has still been controversial [26]. One of the most intriguing questions proposed is whether the electron–phonon coupling (EPC) effect plays a role in superconductivity [27]. As a well-acknowledged unconventional superconductor, the parent compound FeSe exhibits superconductivity at around 8 K [1], which seems to be beyond the framework of BSC theory. However, many time-domain experiments, such as time-resolved X-ray diffraction, photoemission spectroscopy, and ultrafast photocarrier dynamics, reveal that the contributions from the phonons cannot be omitted [23,24,25,28,29]. Surprisingly, the superconducting transition temperature ($T_{c}$) of the two-dimensional FeSe grown on substrates increases to 65 K [30]. This conspicuous increment in $T_{c}$ can be ascribed to the interfaced soft phonon mode between the FeSe layer and the substrates [24]. When applying hydrostatic pressure, the $T_{c}$ of FeSe reaches the maximum value of nearly 40 K [31]. The pressure-dependent behavior of $T_{c}$ was well characterized by theoretical predictions, in which the enhancement of superconductivity is positively correlated with the enhanced EPC interactions induced by the spin fluctuations [32]. This positive correlation was evidenced by the magnetic resonance measurements [33]. Both the theoretical and experimental studies emphasized the importance of the phonons on unconventional superconductivity.

As a member of iron chalcogenides, FeTe${}_{0.5}$Se${}_{0.5}$ has recently become a popular topic of significant interest due to being an ideal platform for realizing quantum computation [34,35,36]. The band structure near the Brillouin zone exhibits an evident inversion induced by the introduction of Te atoms. Although the electronic structure features a remarkable transformation, the $T_{c}$ of FeTe${}_{0.5}$Se${}_{0.5}$ does not change so drastically, slightly increasing to about 15 K at ambient pressure [2]. Upon compression, the $T_{c}$ further increases to a maximum of 25 K [2]. Intriguingly, the pressure-evolution of $T_{c}$ shares similar behavior with the parent compound FeSe. This indicates a universal picture of the pairing mechanism of iron chalcogenides. The conspicuous rising of $T_{c}$ and the multilayer structure in nanoscale also imply broader prospects in the field of superconducting acousto-optic applications [7]. However, unlike FeSe, the role of EPC in FeTe${}_{0.5}$Se${}_{0.5}$ has rarely been discussed. The direct observation of the EPC effect has been absent. Luckily, there are many techniques, such as the time-resolved femtosecond spectroscopy [28,29,37], the wavelength-modulated reflectivity spectroscopy [38], the photon echo spectroscopy [39], and the Raman scattering spectroscopy [37,39,40], that are developed and widely utilized to investigate the dynamics of various excitations. Among these techniques, the time-resolved femtosecond spectroscopy and Raman scattering spectroscopy can be easily combined with the high-pressure techniques, providing excellent insight into the ultrafast dynamics of diverse excitations under hydrostatic pressure, such as hot phonons and electrons, and giving a discernible clue to the pressure evolution of the EPC effect [28,29,37].

In this paper, we carried out pressure-dependent femtosecond spectroscopy and Raman scattering spectroscopy to investigate the EPC effect in FeTe${}_{0.5}$Se${}_{0.5}$. The amplitude of the differential reflectivity ($\frac{\Delta R}{R}$) undergoes a drastic increment before 2.2 GPa, followed by a sudden reduction at higher pressure. The abnormality of the $\frac{\Delta R}{R}$ emerging at approximately 2.2 GPa can be attributed to the structural transition from the tetragonal to monoclinic phase. The amplitude of the electrons and LO phonons term also exhibits similar abnormality at this critical pressure. Notably, the lifetime of the fast-decay component of electrons decreases up to 2.2 GPa and then prolongs upon further compression, which is in favor of the enhanced EPC strength. Two Raman peaks, belonging to the $A_{1g}$ and $B_{1g}$ phonon mode of the tetragonal phase, can be observed within the whole pressure range. Above 2.3 GPa, a new peak corresponding to the newly formed monoclinic phase appears. Surprisingly, the $A_{1g}$ phonon mode displays an obvious softening at approximately 2.3 GPa. The soft phonon mode is a fingerprint of the enhancement of the EPC effect, consistent with the ultrafast photocarrier dynamics. Our work gives an insightful understanding of the role of phonons in iron chalcogenides.

## 2. Materials and Methods

The high-quality single-crystal FeTe${}_{0.5}$Se${}_{0.5}$ bulk, with a cylindrical shape of approximately 0.8 cm in diameter and 0.5 cm in height, was grown by the unidirectional solidification method [41,42] at Brookhaven National Laboratory. The oxygen-annealing method [42] was employed to wipe off the interstitial Fe atoms. Our previous study reveals that the sample is well grown with negligible Fe atoms at the interstitial site [43]. To perform the high-pressure experiments, the bulk sample was dissociated and cleaved into shiny and thin pieces with a size of approximately 100 × 100 µm and a thickness of approximately 40 µm, and then loaded into the diamond anvil cell (DAC). A stainless-steel circle gasket was used, in which a hole with a diameter of approximately 300 µm was made by laser to hold the sample chamber. The argon was used as the transmission medium. A ruby granule was loaded jointly beside the sample to calibrate the pressure. The high-pressure experiments in this study were all carried out at room temperature.

**Figure 1:**
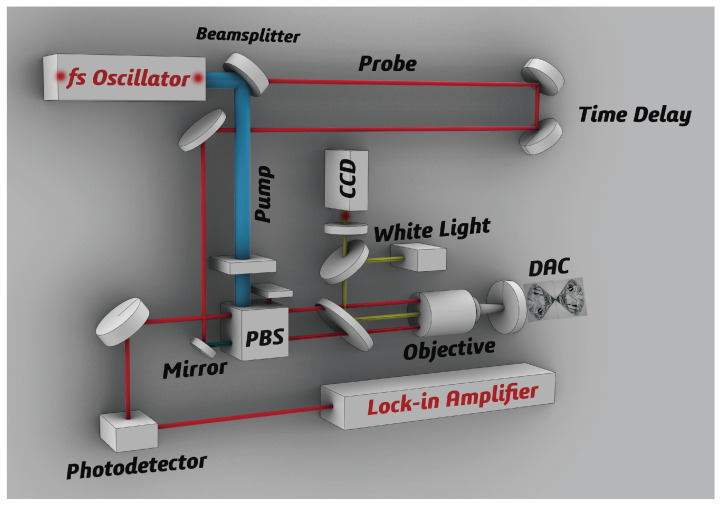
Schematic blocks of the high-pressure femtosecond spectroscopy setup. The laser is separated into pump and probe beamlines. The probe beamlines undergo a time delay. The block PBS denotes the polarized beam splitter.

The high-pressure femtosecond pump-probe measurements were carried out at Hefei Key Laboratory using DAC with a culet of 500 µm. For clarity, the schematic illustration of the femtosecond experiments and the process of obtaining the differential reflectivity is depicted in Figure 1. The 800 nm pulse laser used in femtosecond spectroscopy, with a pulse duration of 150 fs and a repetition rate of 5.2 MHz, was excited by a Ti:sapphire femtosecond oscillator. Two beamlines, the pump and probe laser, were separated from the laser source. The fluency of the pump laser was set to be 370 µJ/cm${}^{2}$, 5 times larger than that of the probe laser. The pump beamlines passed through a polarized beam splitter (PBS) and were then focused into the DAC. The probe beamlines underwent a time-delay stage and were then reflected into the objective and DAC together with the pump beamlines. After contacting the sample surface in DAC, the signal of the reflected probe beamlines passed through the PBS again and was collected by a silicon photodetector equipped with a lock-in amplifier. In ultrafast femtosecond dynamics, the electronic excitations were ignited by a pump laser and transferred their energy to the longitudinal-optical (LO) or longitudinal-acoustic (LA) phonons. In the process of energy decay, the EPC strength can be directly obtained by extracting the lifetime of the excited particles from the differential reflectivity.

The Raman scattering measurements were carried out in Shanghai HPSTAR, using a low-fluorescence DAC with a culet of 500 µm. The Raman scattering spectra were activated using a sapphire laser system with a wavelength of 488 nm, and collected by single-stage spectrograph equipment. An 1800 lines/mm grating was used to split the backscattering light. The incident power of the laser source was set to be 2.5 mW with a spot diameter of approximately 10 µm.

## 3. Results and Discussion

### 3.1. High-Pressure Femtosecond Spectroscopy

**Figure 2:**
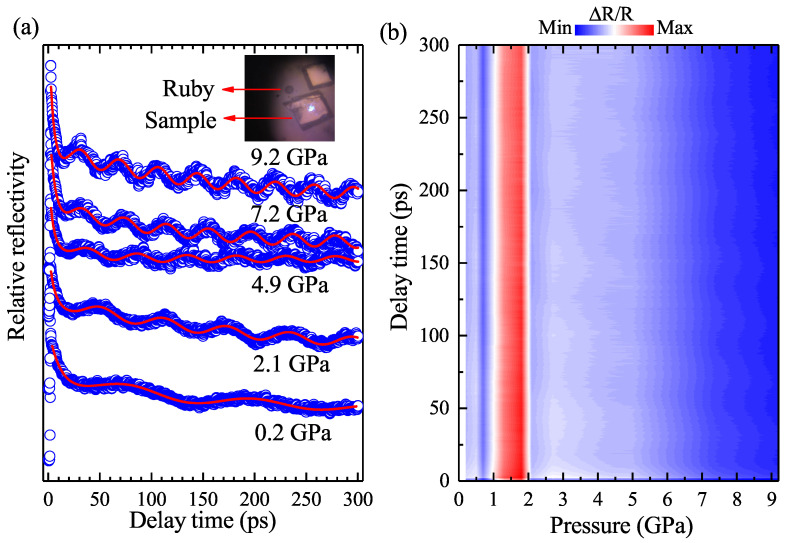
Pressure-dependent femtosecond spectroscopy of FeTe${}_{0.5}$Se${}_{0.5}$ from the pressure range of 0.2 to 9.2 GPa. (**a**) Normalized time-resolved differential reflectivity $\frac{\Delta R}{R}$ at different pressures. The inset figure on the top shows the loaded ruby and two pieces of shiny samples. The blue open circles denote the experimental data measured at different pressures. The red solid lines represent the fitting curves. (**b**) Mapping of the pressure-dependent femtosecond spectroscopy. The spectrum intensity of the $\frac{\Delta R}{R}$ is presented by different colors placed at the top of the figure.

To investigate the performance of the EPC effect, we carried out pressure-dependent femtosecond spectroscopy on a single-crystal FeTe${}_{0.5}$Se${}_{0.5}$ from 0 to 9.2 GPa. The obtained differential reflectivity $\frac{\Delta R}{R}$ at different pressures is normalized and displayed in Figure 2a. For clarity, only five representative curves are displayed in this figure. A phenomenological equation was used to delineate the relaxation process [29]:

(1) $$\frac{\Delta R}{R}=A_{e}e^{-t/\tau_{e}}+A_{LO}e^{-t/\tau_{LO}}+A_{0}+A_{LA}e^{-t/\tau_{LA}}\sin\left[2\pi t/T\left(t\right)+\phi \right],$$

The electrons are excited in a very short time and then release their energy with a much prolonged lifetime. The first term of Equation (1) describes the relaxation process of the excited electrons, with the $A_{e}$ and $\tau_{e}$ denoting the initial population and lifetime, respectively. The second term belongs to the LO phonons consisting of a phonon number $A_{LO}$ and relevant lifetime $\tau_{LO}$. The third term $A_{0}$ is the energy dissipation to the environment background and can be considered as a constant. The last term describes the decay of LA phonons, which is parameterized by the phonon numbers $A_{LA}$ and corresponding lifetime $\tau_{LA}$. The exponential decay process of the LA phonons is superimposed with a sinusoidal oscillation term, with T(t) and ϕ denoting the period and initial phase, respectively. As the pressure increases, the period of the oscillation is manifestly shortened. The fitting matches well with our experimental data.

Figure 2b displays the color map of the pressure-dependent $\frac{\Delta R}{R}$ as a function of the delay time. The reflectivity shows a positive response within the whole delay time of 300 ps and the whole pressure range up to 9.2 GPa. The intensity of the reflectivity spectrum is displayed by a color bar on the top of the figure. There appears to be an evident amplitude change around 2.2 GPa. Above 2.2 GPa, the amplitude continues to decrease with the applied pressure. From Equation (1), we know that the amplitude of the $\frac{\Delta R}{R}$ reflects the proportion to the density of the excited states [29]. Therefore, there must be something interesting happening around this critical pressure point, which will be quantitatively analyzed later.

**Figure 3:**
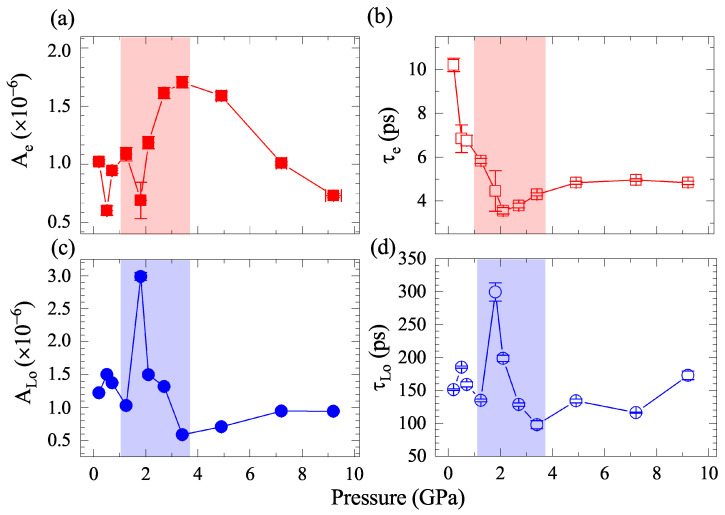
Pressure dependence of the amplitude (**a**) $A_{e}$, (**c**) $A_{LO}$ and the relaxation time (**b**) $\tau_{e}$, (**d**) $\tau_{LO}$. The red symbols are the fitting parameters of electrons obtained by Equation (1), while the blue symbols denote those of the LO phonons. The solid and open symbols denote the amplitude and relaxation time of the fitting results, respectively.

Using Equation (1), we obtained detailed information about the decay process. The fitting results of excitations are summarized in Figure 3a–d. For the fast-decay component of electrons, the amplitude $A_{e}$ first shows an abnormal increment above 2.2 GPa and then decreases when pressurized to 3.4 GPa. The lifetime of the excited electrons ($\tau_{e}$) shortens from 0.2 to 2.2 GPa and prolongs when further pressurizing. For comparison, the $\tau_{e}$ of FeTe${}_{0.5}$Se${}_{0.5}$ obtained in this study (3.5–10 ps) is larger than that of the parent compound FeSe (1.5–2 ps) [29] and single-layer FeSe on substrates (0.23 ps) [24]. As indicated by previous research, the fast component of ultrafast dynamics is the fingerprint of the EPC strength [29,37,44]. The correlations between $\tau_{e}$ and EPC strength can be written as:

(2) $$\lambda \langle \omega^{2}\rangle =\frac{2\pi }{3}\frac{k_{B}T_{e}}{\hbar \tau_{e}},$$

Here, $\lambda \langle \omega^{2}\rangle$ is the second moment of the Eliashberg function, $T_{e}$ is the electronic temperature (which can be approximated as room temperature), and $\langle \omega^{2}\rangle$ is the square frequency of vibrational modes. In iron chalcogenides, one special vibrational mode, the $A_{1g}$ phonon mode, contributes more significantly to the EPC effect than the others [29,32,45,46]. Both the theoretical calculations and experiments affirm that the $A_{1g}$ phonon mode dominates the electron–phonon spectral function. The role of phonons will be investigated and discussed in the following part on the Raman scattering experiments. As described by Equation (2), the $\lambda \langle \omega^{2}\rangle$ is inversely proportional to the lifetime of electrons. This means that the EPC strength is profoundly enhanced at the critical pressure of approximately 2.2 GPa.

For the slow-decay component of LO phonons, both the amplitude and lifetime of the $A_{LO}$ component feature a sudden increase at around 2 GPa, followed by a leveling off at higher pressure. Previous studies revealed that the increase in phonon lifetime is closely related to the well-known bottleneck effect [37,44,47] which is induced by the gap shrinkage when entering the superconducting state. In the context of the phonon bottleneck effect, the amplitude diminishes concurrently with the increasing phonon lifetime. However, the pressure evolution of phonon amplitude we observed at around 2.2 GPa behaves in the opposite way. Therefore, the two remarkable changes in the LO phonons’ amplitude and lifetime cannot be attributed to the phonon bottleneck effect. Other reasons should be found to explain this phenomenon. Previous X-ray diffraction experiments uncovered that the FeTe${}_{0.5}$Se${}_{0.5}$ undergoes a structural transition from a tetragonal to monoclinic phase at around 2.4 GPa [18]. From the crystal symmetry analysis, we know that the symmetry of the monoclinic phase is greatly reduced compared with the tetragonal phase. In the relaxation process of ultrafast dynamics, the LO phonons transfer their energy to the lattice and then degenerate into LA phonons [29]. Accordingly, it is reasonable to speculate that the anomaly of the LO phonon amplitude has a potential connection with the structural transformation, during which the energy propagation to lower energy excitations might be hindered by symmetry devastation. As a consequence, the high-energy phonons accumulate gradually, resulting in an increased amplitude and elongated lifetime.

### 3.2. High-Pressure Raman Scattering Spectroscopy

**Figure 4:**
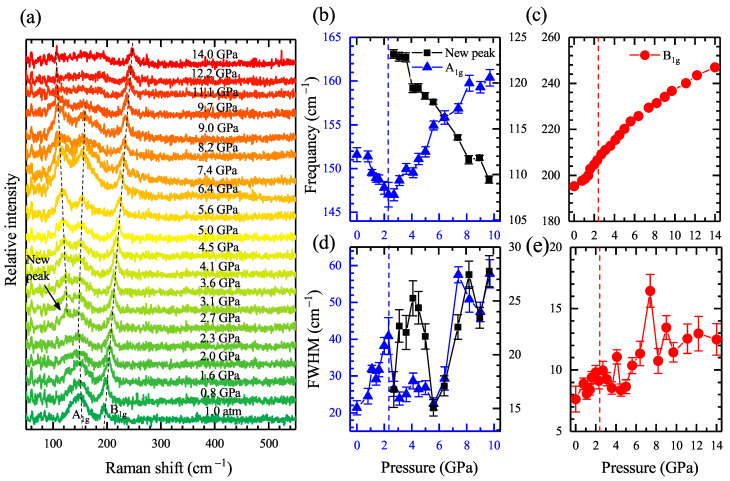
Pressure-dependent Raman scattering spectroscopy FeTe${}_{0.5}$Se${}_{0.5}$. (**a**) Experimental data in the pressure range from 0 to 14 GPa. The solid lines with gradient ramps are the curves of the raw data at different pressures. Two Raman active modes, the $A_{1g}$ and $B_{1g}$ phonon modes, are observed. Above 2.3 GPa, a new peak with a wavelength of approximately 123 cm${}^{-1}$ appears, marked by a black arrow. The tracks of the positions of the three peaks are marked by black dash lines. (**b**–**e**) Pressure dependence of the frequency (**b**,**c**) and FWHM (**d**,**e**). The red and blue symbols denote the fitting results of the $A_{1g}$ and $B_{1g}$ modes, respectively. The black symbols denote the fitting results of the new peak.

To gain further insight into the EPC effect in FeTe${}_{0.5}$Se${}_{0.5}$, we performed pressure-dependent Raman scattering measurements from ambient pressure up to 14 GPa. Figure 4a demonstrates the experimental data of the high-pressure Raman spectroscopy after subtracting the baseline. Two vibrational modes at 152 and 196 cm${}^{-1}$ at 0 GPa are observed, which can be assigned to the $A_{1g}$ and $B_{1g}$ modes of the tetragonal phase, respectively [18,45,48,49]. Looking at the figure, one can find that the frequency of the $A_{1g}$ mode evidences a softening before 2.3 GPa, followed by a hardening behavior. The frequency of the $B_{1g}$ mode displays a robust blueshift as the pressure increases to 14 GPa.

**Figure 5:**
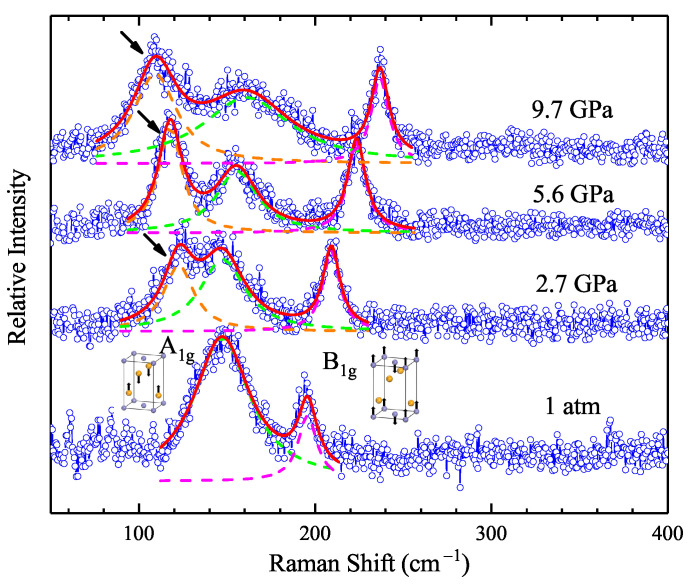
Peak splitting and fitting process of the pressure-dependent Raman scattering spectroscopy. The blue open symbols and red solid lines denote the experimental data and the fitting results, respectively. The green-, purple-, and yellow-dashed lines represent the peak fitting of the $A_{1g}$, $B_{1g}$, and the new phonon modes, respectively. The two inset pictures are the atomic-displacement patterns of Raman-active modes, the $A_{1g}$ and $B_{1g}$ modes, respectively. The blue atoms are the Fe atoms, while the yellow ones are the Se/Te atoms. The peak of the new phonon mode is marked by the black arrow. For clarity, four representative curves at different pressures were selected and displayed in this figure.

We use a Lorentzian function [49] to fit the curves and extract the pressure-dependent frequency and linewidth of the three observed phonon modes, as demonstrated in Figure 4b–e. The fitting curves are displayed in Figure 5 and they match well with the experimental data. The softening of about 6 cm${}^{-1}$ for the $A_{1g}$ mode at about 2.3 GPa can now be precisely witnessed. Meanwhile, the phonon linewidth reaches a local maximum value of 40 cm${}^{-1}$ at around 2.3 GP, decreases, and then increases with pressure above 5.6 GPa. Theoretically, the EPC constant can be characterized by Equation (3):

(3) $$\lambda N\left(0\right)=\frac{1}{2\pi }\frac{\gamma }{\omega^{2}},$$

where N(0) is the density of states at the Fermi level, γ is the phonon linewidth, and ω is the phonon frequency. Since the EPC constant is proportional to the γ and inversely proportional to the square of ω, the opposite behaviors of these two parameters indicate an enhancement of the EPC strength at the critical pressure point of 2.3 GPa. The frequency of the $B_{1g}$ hardens with increasing pressure, accompanied by the general broadening of the phonon linewidth. The new peak emerging above 2.3 GPa is also fitted by the Lorentz function, and the results are inset in Figure 4a,c. Contrary to the $A_{1g}$ and $B_{1g}$ mode, the new Raman active mode has been softening with applied pressure since it appeared, while the phonon linewidth shares similar pressure evolution with the $A_{1g}$ mode.

As shown in Figure 4a, a new peak at 123 cm${}^{-1}$ emerges when reaching the critical pressure point of approximately 2.3–2.7 GPa, and softens with further compression. For better illustration, we displayed the representative Raman spectrum at four different pressures with the relevant fitting process in Figure 5. The new Raman active mode appearing above 2.3 GPa can now be observed distinctively. The fitting matches well with our experimental data. In this case, one would wonder what ignites the peak splitting of the phonon modes above 2.3 GPa. Preceding Raman scattering studies on the antiferromagnetic parent compound FeTe with monoclinic crystal structure found a Raman active mode at about 120 cm${}^{-1}$, close to that of our observation [50]. According to previous X-ray diffraction experiments, the sample FeTe${}_{0.5}$Se${}_{0.5}$ undergoes a phase transition from a tetragonal to monoclinic phase at around 2.3 GPa [18]. Therefore, we conclude that this new peak is a token of the high-pressure monoclinic crystal structure, consistent with our high-pressure femtosecond experiments.

### 3.3. Dual Evidence for the Enhancement of the Electron-Phonon Coupling Effect

**Figure 6:**
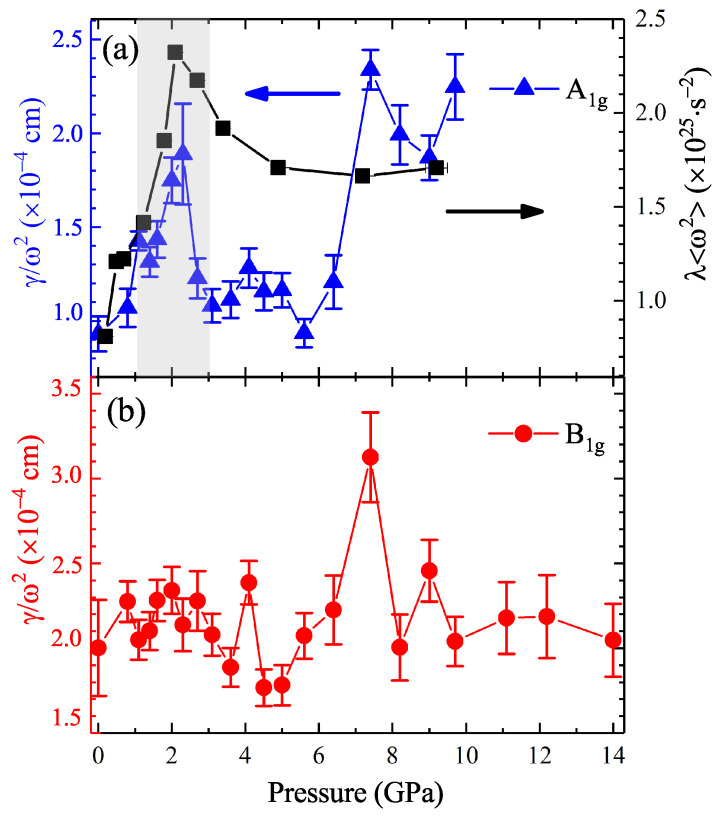
Pressure-dependent parameters of the electron–phonon coupling strength. The blue and red symbols denote the electron–phonon coupling constants obtained from the vibrational properties of the (**a**) $A_{1g}$ and (**b**) $B_{1g}$ phonon modes. The black symbols plotted are the calculated second term of the Eliashberg function. Figure (**a**) is plotted as a double-Y-axis form.

The calculated EPC constant using Equation (3) and the second term of the Eliashberg function obtained by Equation (2) are summarized in Figure 6. The coupling strength of the $A_{1g}$ mode demonstrates a sharp peak at approximately 2.3 GPa. Simultaneously, the second term of the Eliashberg function that reflects the EPC strength also exhibits a salient peak near this critical point. As the pressure further increases, both terms show an increasing trend above 5.6 GPa. Previous theoretical predictions and ultrafast dynamics revealed that the $A_{1g}$ mode is the strongest component in the EPC spectroscopy of iron chalcogenides [29,32,45,46]. When applying pressure, the superconductivity is enhanced by the spin fluctuations induced by the $A_{1g}$ phonon. Therefore, the consistent behavior of the two pressure-dependent parameters provide compelling evidence for the enhancement of the EPC effect in FeTe${}_{0.5}$Se${}_{0.5}$. For the $B_{1g}$ mode, the EPC constant is almost independent of pressure, except for an abrupt argument appearing above 5.6 GPa. Our results emphasized the importance of the $A_{1g}$ phonon mode on the interactions between electrons and phonons.

When comparing with the previously reported results of the pressure-dependent $T_{c}$ [2,18,51], we found a positive correlation between the EPC strength and the superconducting temperature. As reported by Gresty et al., Horigane et al., and Tsoi et al., the $T_{c}$ increases with pressure and reaches a maximum value at around 2.3–2.5 GPa. The enhancement of $T_{c}$ is positively correlated with the increasing EPC strength obtained from the $A_{1g}$ phonon and the ultrafast femtosecond dynamics, indicating the potential role of the EPC effect on superconductivity. It must be emphasized that the high-pressure experiments in this paper were carried out at room temperature. If one wants to explore the high-pressure femtosecond and Raman scattering spectroscopy in the superconducting state, much more effort needs to be made, which includes realizing the in situ pressurization and constructing a cryogenic vacuum chamber. Nevertheless, our results provide excellent guidance for high-pressure and low-temperature experiments in the future. We do expect even more interesting physics at low temperatures.

## 4. Conclusions

In summary, we have investigated the effect of pressure on the ultrafast photocarrier dynamics and vibrational properties of FeTe${}_{0.5}$Se${}_{0.5}$. The structural transition and the coupling effect between the electrons and phonons under pressure have been systematically studied. We confirmed the structural transition from a tetragonal to monoclinic phase at 2.2–2.3 GPa by the abnormal amplitude change of the longitudinal-optical phonons and the appearance of a new Raman active mode. The lifetime of excited electrons displays a reduction at 2.2 GPa. Meanwhile, the $A_{1g}$ mode exhibits an unusual frequency softening along with the concurrently increasing phonon linewidth. Both femtosecond and Raman scattering spectroscopy unveil the prominent enhancement of the electron–phonon coupling strength at 2.2–2.3 GPa. The misalignment of the critical pressure of less than 0.1 GPa for the two experiments is within the error of the pressure calibration. Furthermore, our results imply that it is the $A_{1g}$ that contributes the most to the electron–phonon coupling, rather than the $B_{1g}$ mode. Our work provides an insightful perspective on the pressure effects of the ultrafast photocarrier dynamics and gives a comprehensive understanding of the electron–phonon coupling effect in FeTe${}_{0.5}$Se${}_{0.5}$. The understanding of the role of phonons in superconductivity might also help the development of applications of superconducting acousto-optic devices, such as superconducting radio-frequency cavities, in the foreseeable future.

## Data Availability

Not applicable.

## Abbreviations

The following abbreviations are used in this manuscript:

DAC	Diamond anvil cell
EPC	Electron–phonon coupling
$T_{c}$	Superconducting transition temperature
LO	Longitudinal optic
LA	Longitudinal acoustic
